# Understanding the role of potential biomarkers in attenuating multiple sclerosis progression via multiomics and network-based approach

**DOI:** 10.1371/journal.pone.0314428

**Published:** 2024-12-19

**Authors:** Nitesh Shriwash, Ayesha Aiman, Prithvi Singh, Seemi Farhat Basir, Anas Shamsi, Mohammad Shahid, Ravins Dohare, Asimul Islam

**Affiliations:** 1 Centre for Interdisciplinary Research in Basic Sciences, Jamia Millia Islamia, Okhla, New Delhi, India; 2 Department of Biosciences, Faculty of Natural Sciences, Jamia Millia Islamia, Okhla, New Delhi, India; 3 Centre of Medical and Bio-allied Health Sciences Research, Ajman University, Ajman, United Arab Emirates; 4 Department of Basic Medical Sciences, College of Medicine, Prince Sattam Bin Abdulaziz University, Al-Kharj, Saudi Arabia; Dasman Diabetes Institute, KUWAIT

## Abstract

**Background:**

Multiple sclerosis (MS) is a complex neurological disorder marked by neuroinflammation and demyelination. Understanding its molecular basis is vital for developing effective treatments. This study aims to elucidate the molecular progression of MS using multiomics and network-based approach.

**Methods:**

We procured differentially expressed genes in MS patients and healthy controls by accessing mRNA dataset from a publicly accessible database. The DEGs were subjected to a non-trait weighted gene co-expression network (WGCN) for hub DEGs identification. These hub DEGs were utilized for enrichment, protein-protein interaction network (PPIN), and feed-forward loop (FFL) analyses.

**Results:**

We identified 880 MS-associated DEGs. WGCN revealed a total of 122 hub DEGs of which most significant pathway, gene ontology (GO)-biological process (BP), GO-molecular function (MF) and GO-cellular compartment (CC) terms were assembly and cell surface presentation of N-methyl-D-aspartate (NMDA) receptors, regulation of catabolic process, NAD(P)H oxidase H_2_O_2_ forming activity, postsynaptic recycling endosome. The intersection of top 10 significant pathways, GO-BP, GO-MF, GO-CC terms, and PPIN top cluster genests identified *STAT3* and *CREB1* as key biomarkers. Based on essential centrality measures, *CREB1* was retained as the final biomarker. Highest-order subnetwork FFL motif comprised one TF (*KLF7*), one miRNA (miR-328-3p), and one mRNA (*CREB1*) based on essential centrality measures.

**Conclusions:**

This study provides insights into the roles of potential biomarkers in MS progression and offers a system-level view of its molecular landscape. Further experimental validation is needed to confirm these biomarkers’ significance, which will lead to early diagnostic and therapeutic advancements.

## 1. Introduction

In 2020, the global health challenge gained alarming momentum. Multiple Sclerosis (MS), a multifaceted neurological disorder, witnessed a significant surge in prevalence, reaching 2.8×10^6^ individuals worldwide. This represented a staggering 30% increase from the data recorded in 2013 [1]. These numbers paint a stark picture of a condition on the rise, but what lies behind these statistics is a complex and often unpredictable disease that affects not only the individuals it strikes but also their families, friends, and the broader healthcare system.

MS primarily targets the central nervous system (CNS) [2, 3], including the brain and spinal cord, which collectively govern crucial bodily functions. At its core, MS is characterized by the deterioration of the protective myelin sheath surrounding nerves- a bit like the insulation on an electric wire. This process of demyelination disrupts the normal transmission of electrical signals to and from the brain, resulting in a diverse array of debilitating symptoms. These symptoms include blurred vision, muscular weakness, tingling sensations, dizziness, and overwhelming fatigue. There are three clinical courses observed in MS [4]. The most prevalent form is relapsing-remitting multiple sclerosis (RRMS), constituting approximately 85% of cases [5]. In RRMS, individuals experience cycles of relapse followed by remission, with symptoms ranging from mild to severe. After an indeterminate period, a majority of those with RRMS transition to secondary-progressive multiple sclerosis (SPMS), marked by ongoing neurological decline without remission intervals. In contrast, about 15% of individuals with MS follow a primary progressive multiple sclerosis (PPMS) characterized by a continuous deterioration of neurological functioning without distinct relapses or remissions. While the progression rate in PPMS may exhibit variability over time, occasional plateaus or temporary improvements can occur, but the overall trend remains one of continuous deterioration [4, 6].

Perhaps one of the most perplexing aspects of MS is its substantial heterogeneity among individuals [7, 8]. Some experience phases of relapse and remission, while others face a progressively worsening trajectory. This unpredictability adds to the complexity of the disease, making it a formidable challenge for both those living with MS and the healthcare community. MS doesn’t discriminate by age, but it does have a predilection for striking young adults [9, 10]. Alarmingly, it is recognized as a leading cause of non-traumatic neurological disability in this demographic in numerous countries. Notably, MS has a distinct gender bias, with a prevalence rate twice as high among females compared to males [10]. What’s even more concerning is the increasing number of cases affecting children; over 30000 cases have been identified in children, indicating a substantial rise from previous estimates [11].

This burgeoning prevalence rate and the persistent disparities in healthcare accessibility underscore the need to address this global health challenge in a comprehensive manner. MS, with its intricate web of symptoms and unpredictable course, has far-reaching consequences, affecting not only the individuals but also their families and the broader healthcare system. As the prevalence continues to rise, particularly among young adults and even children, it becomes increasingly crucial to focus on research, awareness, and equitable healthcare access to mitigate the impact of MS on individuals and society as a whole.

Preceding the 1990s, the primary approach to managing MS centered on the mitigation of relapses using systemic corticosteroids and addressing short-term symptom relief. A significant milestone was reached in 1993 and 1996 when the first two injectable interferon beta (IFNβ) therapies were approved [12–14], providing clinicians with a longer-term treatment option aimed at altering the progression of the disease. Subsequently, the landscape of therapeutic options for MS has expanded considerably. New drug targets and other disease-modifying therapies (DMTs) have emerged, offering patients a wider array of treatment choices. Nevertheless, interferon (IFN) therapy continues to hold substantial relevance for numerous patients. It has demonstrated efficacy in terms of reducing relapse rates, delaying disability progression, and diminishing the number of CNS lesions. A wealth of data supports the long-term effectiveness and safety of IFNs in achieving these outcomes [13, 15]. Moreover, IFNs serve as active comparators in clinical trials assessing newer MS therapies, underscoring their significance as a foundational component of MS treatment. While the manuscript emphasizes the advantages of IFN therapy, it is necessary to address its limitations, including potential side effects and challenges that may reduce therapeutic efficacy. Commonly reported adverse effects include influenza-like symptoms, transient biochemical abnormalities, menstrual irregularities, and elevated muscle spasticity [16]. Additionally, IFNβ has been associated with liver injury, which can range from mild serum enzyme elevations to severe cases involving jaundice or, rarely, acute liver failure. Most cases of liver injury resolve following cessation of therapy, although resolution may be delayed by one to two weeks [17]. Other side effects include inflammation at the injection site, fever-like symptoms, cephalgia, and myalgia [13]. Additionally, the development of neutralizing antibodies remains a concern in a few patients, as it may attenuate the therapeutic efficacy of IFN over time [18].

Although the exact cause of MS is believed to include several factors, the use of molecular techniques to enhance its diagnosis and evaluation still lacks promising outcomes. Consequently, there is a growing emphasis on exploring early MS biomarkers in peripheral blood. The discovery of molecular biomarkers in blood holds significant promise for enhancing the diagnosis, care, and treatment of MS [19, 20]. Notably, recent studies have focused on gene profiling in MS to elucidate messenger RNA (mRNA) signatures in tissues, revealing several differentially expressed genes (DEGs) [6, 21]. Alterations in the expression of microRNAs (miRNAs) have been documented in active brain lesions (BLs) obtained from individuals diagnosed with MS. miRNAs have attracted considerable interest in recent years due to their involvement in disease progression and the formation of tumors [22]. These non-coding entities, spanning 22−24 nucleotides, attach themselves to the complementary 3’ or 5’ untranslated region (UTR) sites on target mRNAs. The binding of miRNA leads to the degradation of mRNA, perhaps necessitating inhibition of translation. They are linked to the argonaute protein family, forming a silencing complex that controls multiple pathways. They exhibit pleiotropy, exhibiting many gene targets and vice versa. miRNAs are essential for several biological process (BPs), such as cell cycle regulation, apoptosis induction, angiogenesis control, metabolic regulation, inflammation modulation, immune response (IR) regulation, proliferation regulation, and lung injury management. The disturbance in miRNA expression is linked to various human ailments, including MS. Changes in miRNA levels under abnormal circumstances result in a distinctive pattern, which may serve as a biomarker or molecular target for treating associated diseases. While various cell types exhibit unique miRNA expression profiles, mature miRNAs have been identified in extracellular fluids. Referred to as circulating microRNAs (c-miRNAs), these molecules demonstrate exceptional stability and resilience to unfavourable physiological conditions, such as prolonged storage, repeated freeze-thaw cycles, and pH variations. Consequently, circulating miRNAs emerge as promising candidates for biomarkers. Medications like IFNβ and glatiramer acetate (GA), used in the treatment of MS, may modulate the expression of miRNA, potentially offering advantages to individuals with MS [23].

In neurological disorders like MS, demyelination in the CNS is a key feature. While the regenerative process of remyelination occurs naturally, it is often insufficient in preventing axonal loss and restoring function [24]. The CNS faces challenges in axon regeneration linked to a decline in the intrinsic ability of neurons. The Krüppel-like factor (KLF) family of transcription factor (TFs), particularly KLF Transcription Factor 7 (*KLF7*), plays a role in regulating regenerative potential. *KLF7* expression is developmentally downregulated, but experiments with cortical neurons show that its overexpression promotes axonal growth [25]. This highlights the potential therapeutic role of modulating *KLF7* to enhance axonal regeneration in CNS disorders characterized by demyelination such as MS.

In this manuscript, we have fetched MS-associated DEGs from mRNA expression profile comprising MS and healthy patient samples followed by establishing a weighted gene co-expression network (WGCN) and obtaining hub module and hub DEGs. Later, we associated the hub DEGs with significant pathway and gene ontology (GO) terms followed by protein-protein interaction network (PPIN) construction and modular analysis. Lastly, to elucidate the molecular mechanism and progression pattern behind MS, we dug into the 3-node feed-forward loop (FFL) construction and analysis to identify key miRNA(s) and TF(s) interacting with our hub DEGs(s). Although the present work attempts to capture the portrait of MS molecular mechanism at the systems level from a bioinformatics point of view, further *in-vitro* and *in-vivo* experimentations are still needed in order to validate our findings, which is a limitation of our study. The strength and novelty of the present work is the combined utilization of WGCN, enrichment, PPIN, and FFL analyses which manage to pick up *KLF7*, CAMP responsive element binding protein 1 (*CREB1)*, miR-328-3p responsible for MS progression and may prove to be robust diagnostic as well as predictive biomarkers for its early diagnosis and treatment. This is first of its kind study where the MS progression can be understood from a bioinformatics point of view using a comprehensive multiomics approach.

## 2. Materials and methods

### 2.1. MS-associated mRNA data extraction and differential expression analysis (DEA)

To retrieve MS-associated mRNA expression profile(s), we used “Multiple Sclerosis” as an appropriate keyword to exhaustively search the National Center for Biotechnology Information-gene expression omnibus (NCBI-GEO) [26] (https://www.ncbi.nlm.nih.gov/geo/) database. The search results were further trimmed down in accordance with the following inclusion criteria: (1) dataset(s) must comprise solely “Homo Sapiens” samples; (2) expression profiling by array must be the type of dataset(s); (3) unprocessed as well as preprocessed files availability for the dataset(s) must be present; (4) submission of the dataset(s) must be within last 5 years (i.e., 2017–2022); (5) there must be both healthy control and MS patient samples availability within the dataset(s); (6) atleast 20 patient samples availability must be present. We excluded any type of case reports, review-based articles, cell-line-based experimental study designs, abstracts, and studies devoid of healthy controls or non-human samples. Post pre-processing, batch-correction was performed using ARSyNseq function via NOISeq R package [27, 28] with unknown batch settings. The steps of probe ID to gene mapping, and duplicacy removal were performed in sequence as discussed previously [29, 30]. An unpaired two-sample t-test was applied between healthy control and MS patient sample groups via limma R package [31]. The DEGs were screened corresponding to a statistical cutoff of p−value<0.05 and |log_2_(fold change)|>0.2.

### 2.2. MS-associated non-trait WGCN formation and hub DEGs identification

The steps of noisy DEG(s) elimination, non-trait WGCN establishment and module(s) assignment were performed in sequence as discussed previously [32] via WGCNA R package [33]. We checked for any possible merging of module(s) with similar high-expression profiles post module eigengene (ME) and MEdissimilarity (MEdiss) computation. The steps of hub module(s) selection and hub DEG(s) identification were performed as discussed previously [34].

### 2.3. Functional enrichment and PPIN modular analysis

Enrichr web-based tool [35, 36] (https://maayanlab.cloud/Enrichr/) was accessed for compilation of top 10 significant (p−value<0.05) pathway and GO terms corresponding to MS-associated hub DEGs obtained from WGCN. We used Reactome, GO-cellular compartment (GO-CC), GO-biological process (GO-BP), and GO-molecular function (GO-MF) libraries to compile GO terms and pathways data. The hub DEGs from WGCN were given as an input to the Search Tool for the Retrieval of Interacting Genes (STRING) v12.0 database [37] (https://string-db.org/) for building a PPIN corresponding to medium confidence (i.e., interaction score>0.4) and visualized via Cytoscape v3.10.1 [38]. We used the Molecular Complex Detection (MCODE) plugin [39] to detect communities within our PPIN. Parameters used in MCODE for selecting the hub PPIN module were discussed previously [40]. CytoNCA [41] and NetworkAnalyzer plugins were used to compute the PPIN centrality measures such as node degree (ND), betweenness, eigenvector, and closeness.

### 2.4. MS-associated 3-node miRNA FFL construction and topological analysis

Primarily, we accessed ChEA3 database [42] (https://maayanlab.cloud/chea3/) in order to acquire significant human TFs (p−value<0.05) interacting with our hub DEGs. Next, we accessed miRWalk v3 database [43] (http://mirwalk.umm.uni-heidelberg.de/) to fetch miRNAs interacting with our hub DEGs and acquired TFs as per the following criteria: miRNAs must be binding only on 3’UTR with binding gap = 1 and score ≥ 0.95. All the miRNAs and TFs were validated via literature, and only the ones having an association with MS were retained. All the pairs (i.e., miRNA-TF, miRNA-mRNA, mRNA-TF) were thereafter merged as a union to establish MS-associated 3-node miRNA FFL and visualized via Cytoscape. CytoNCA and NetworkAnalyzer plugins were used to compute the centrality measures of MS-associated 3-node miRNA FFL.

## 3. Results

### 3.1. MS-associated mRNA data extraction and DEA

**Fig 1:**
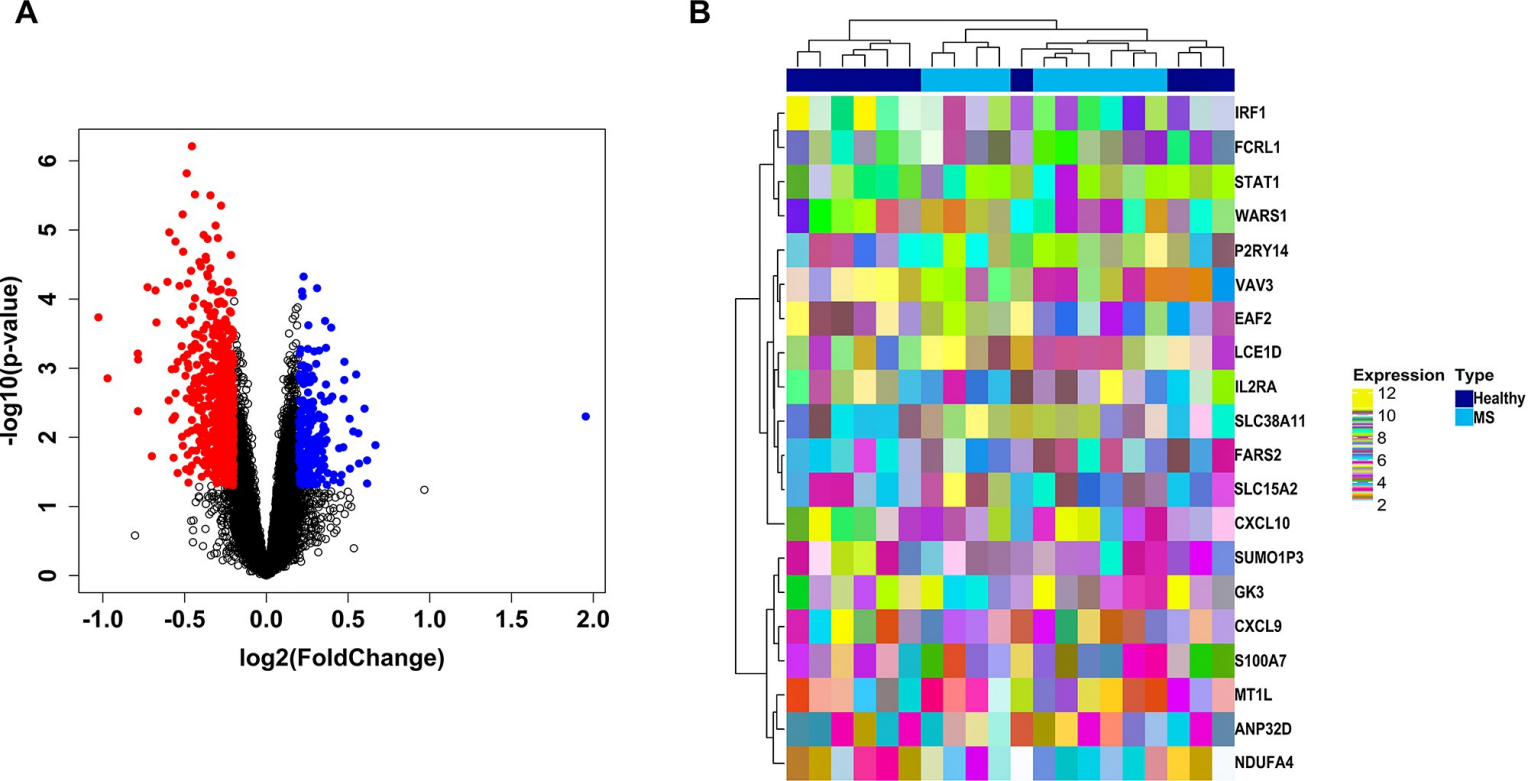
(A) Volcano plot showing the distribution of MS-associated DEGs (red and green dots indicate upregulation and downregulation) and nonsignificant genes (grey dots). (B) Annotation heatmap showing the expression distribution of top MS-associated DEGs (up and downregulated) across MS and healthy patient samples. Left and top sides of the plot signify cluster dendrograms demonstrating Euclidean distance-based hierarchical clustering for columns and rows. Sample-type annotation bars are displayed at the top of heatmap.

As per the above mentioned exclusion and inclusion criteria we picked GSE117935 comprising 10 MS and 10 healthy patient samples. A total of 880 MS-associated DEGs were identified corresponding to abovementioned threshold. The list of DEGs is shown in **S1 Table**. Volcano plot showing the significant (colored dots) and nonsignificant (black-colored points) genes is shown in **Fig 1A**. The expression heatmap showing top 20 MS-associated DEGs (10 up and 10 downregulated) across MS and healthy patient samples is shown in **Fig 1B**. The most significantly down and upregulated DEGs were solute carrier family 38 member 11 (*SLC38A11*) [log_2_(fold change) = −1.02] and C-X-C motif chemokine ligand 10 (*CXCL10*) [log_2_(fold change = 1.95].

### 3.2. MS-associated non-trait WGCN formation and hub DEGs identification

**Fig 2:**
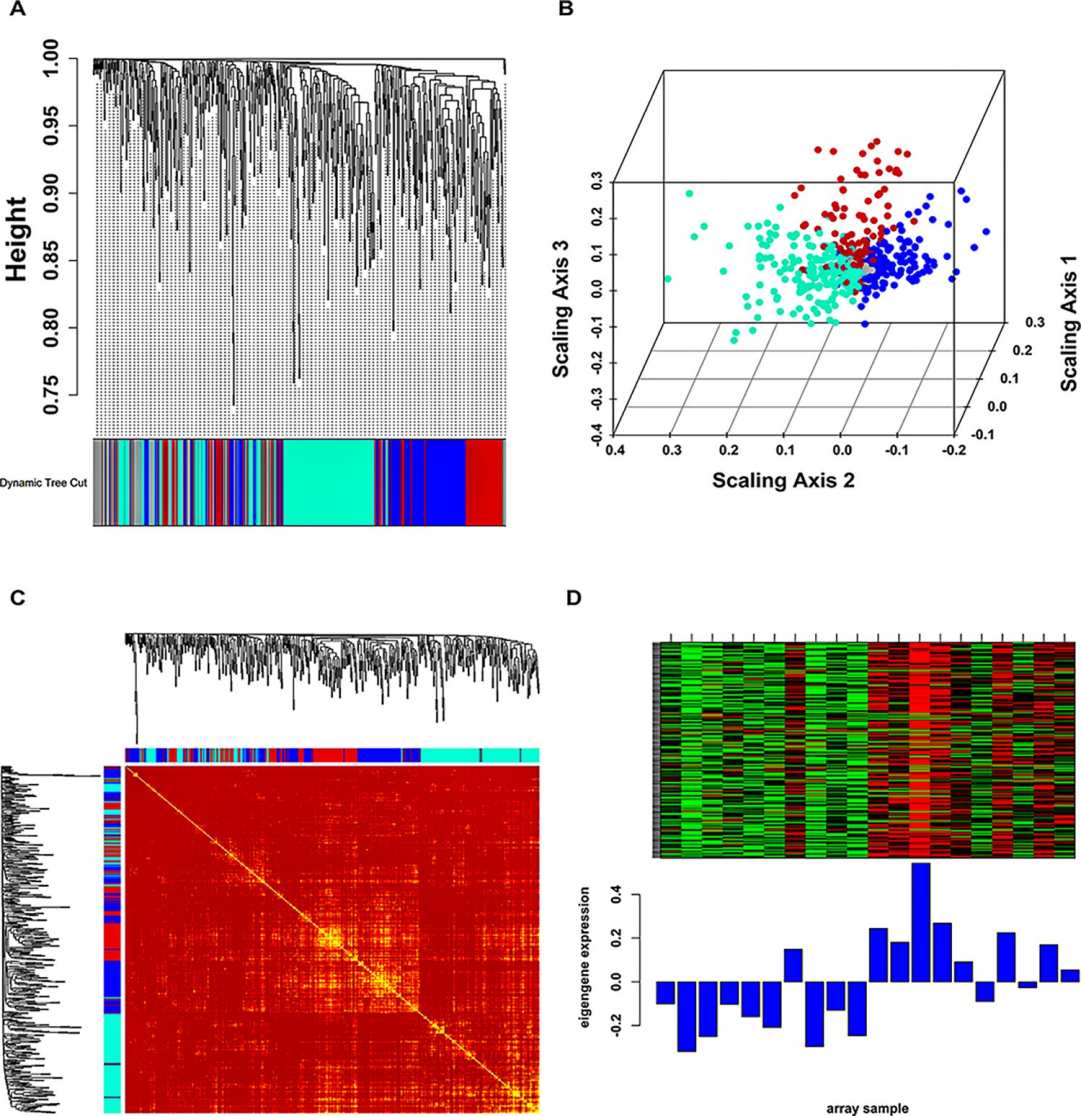
(A) Hierarchical clustering dendrogram of 658 MS-associated DEGs clustered on the basis of dissTOM and four color-coded communities (i.e., blue, turquoise, grey, brown). (B) MDS plot where each colored point signify a gene belonging to a respective color-coded community. (C) Heatmap plot signifying TOM among blue, turquoise, brown community genes. Plot’s left and top side panels signify community assignments and hierarchically clustered gene dendrograms. Dark-colored blocks along the diagonal represent communities. (D) Expression heatmap of blue community genes, wherein the columns and rows relate to samples and genes. The red- and green-colored bands in the heatmaps signify higher and lower expression levels, respectively. Also, the corresponding ME expression levels (y-axis) across the samples (x-axis) are represented at the base panel of each community heatmap as bar plots.

We excluded all the noisy DEGs and utilized a set of 658 non-noisy MS-associated DEGs for WGCN formation. Furthermore, the post-outlier validation quality check (QC) showed that a total of 19 samples were retained out of the initial set of 20. Since our WGCN did not follow the scale-free topology (SFT) criterion, therefore we chose β = 18 (for less than 20 samples) in accordance with the WGCNA guidelines. A total of four color-coded modules were revealed by dynamic tree cut (DTC) algorithm and clustering tree (hierarchical) as shown in **Fig 2A**. **Fig 2B** shows a multidimensional scaling (MDS) plot of all modules. Grey module was discarded for further analysis since it comprised unassigned genes. WGCN representation in the form of a topological overlap matrix (TOM) is shown in **Fig 2C**. Based on module membership (MM) and standard intramodular connectivity (k.in) correlation data (**Table 1**), we picked blue module as our hub module. **Fig 2D** shows a heatmap plot of blue module genes along with their corresponding ME levels. Lastly, a total of 122 hub DEGs were screened from our hub module corresponding to MM > 0.8.

**Table 1:** MM vs k.in correlation and p-values for MS associated modules.

Module	No. of genes	MM vs k.in (correlation)	MM vs k.in (p-value)
**Blue**	179	0.9	9.8×10^−66^
**Turquoise**	225	0.85	5.3×10^−64^
**Brown**	159	0.87	4.4×10^−50^

### 3.3. Functional enrichment and PPIN modular analysis

**Fig 3:**
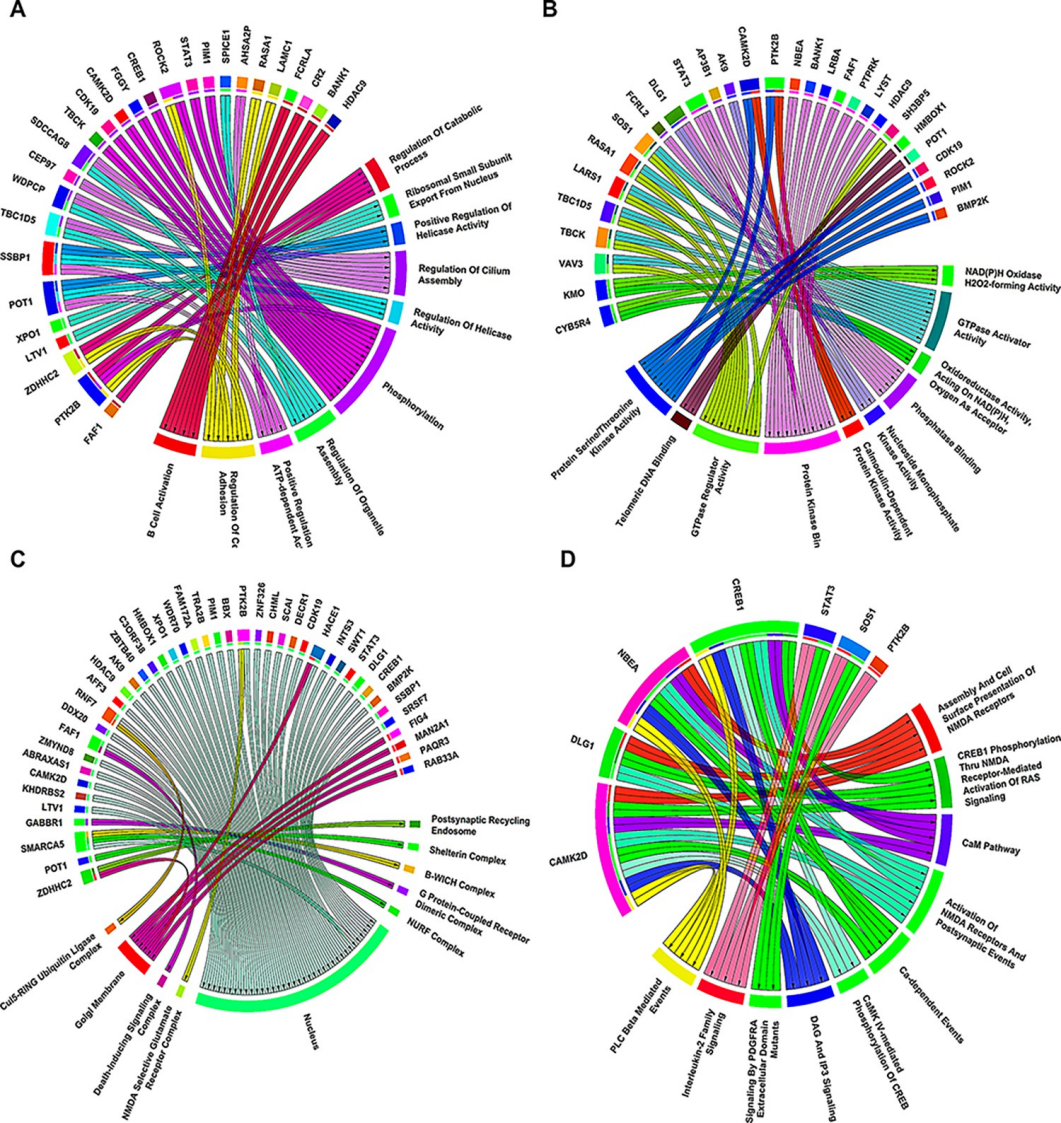
Chord plots demonstrating the association of hub DEGs with top 10 significant (A) GO-BP, (B) GO-MF, (C) GO-CC, (D) pathway terms via undirected and unweighted colored edges.

**Fig 4:**
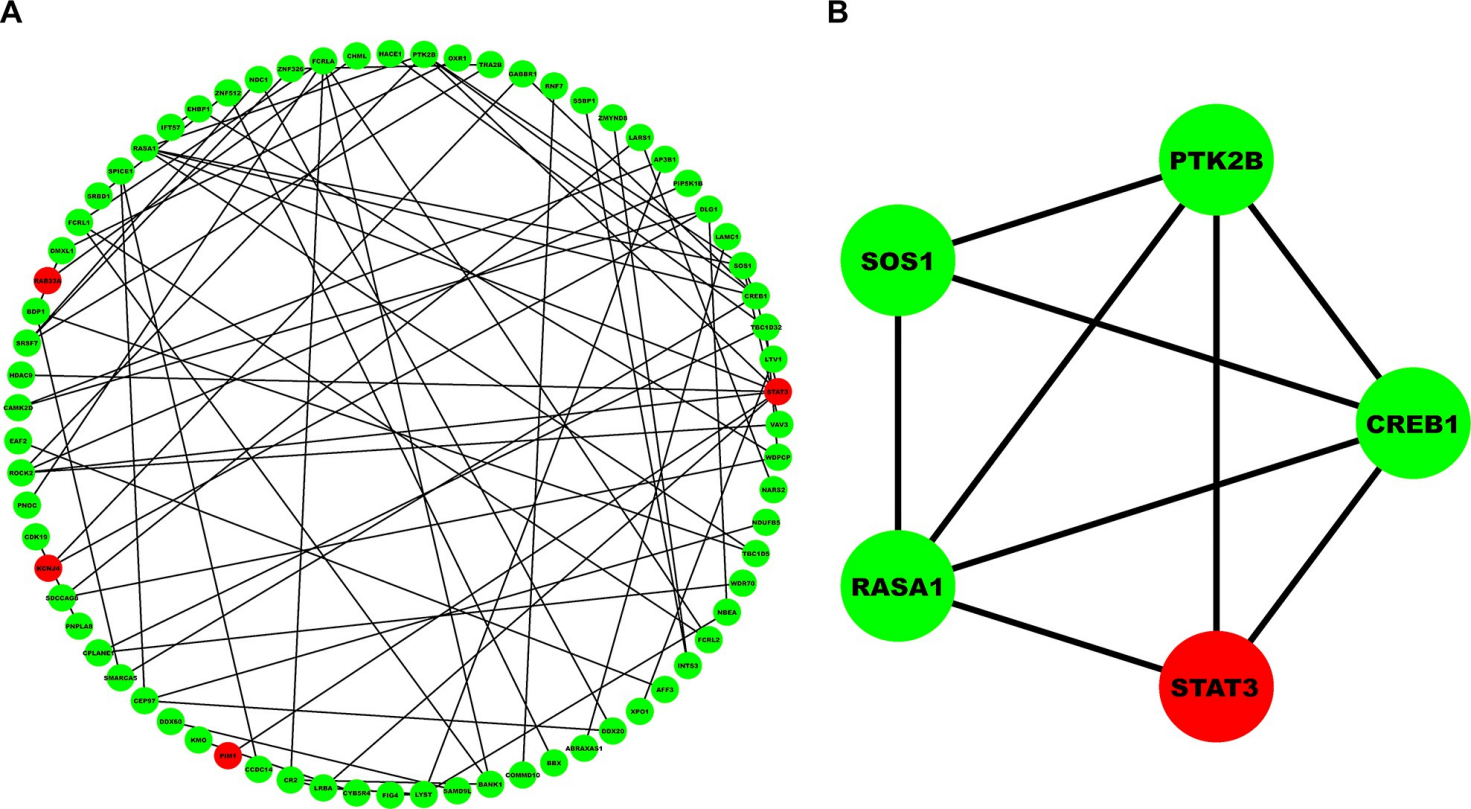
(A) Unweighted and undirected PPIN comprising 71 nodes and 72 edges corresponding to an interaction score >0.4. (B) Highest-scoring PPIN hub module (MCODE score = 4.5) comprising 5 nodes and 9 edges. Red and green colored nodes signify up and downregulated expression status of DEGs.

**Fig 5:**
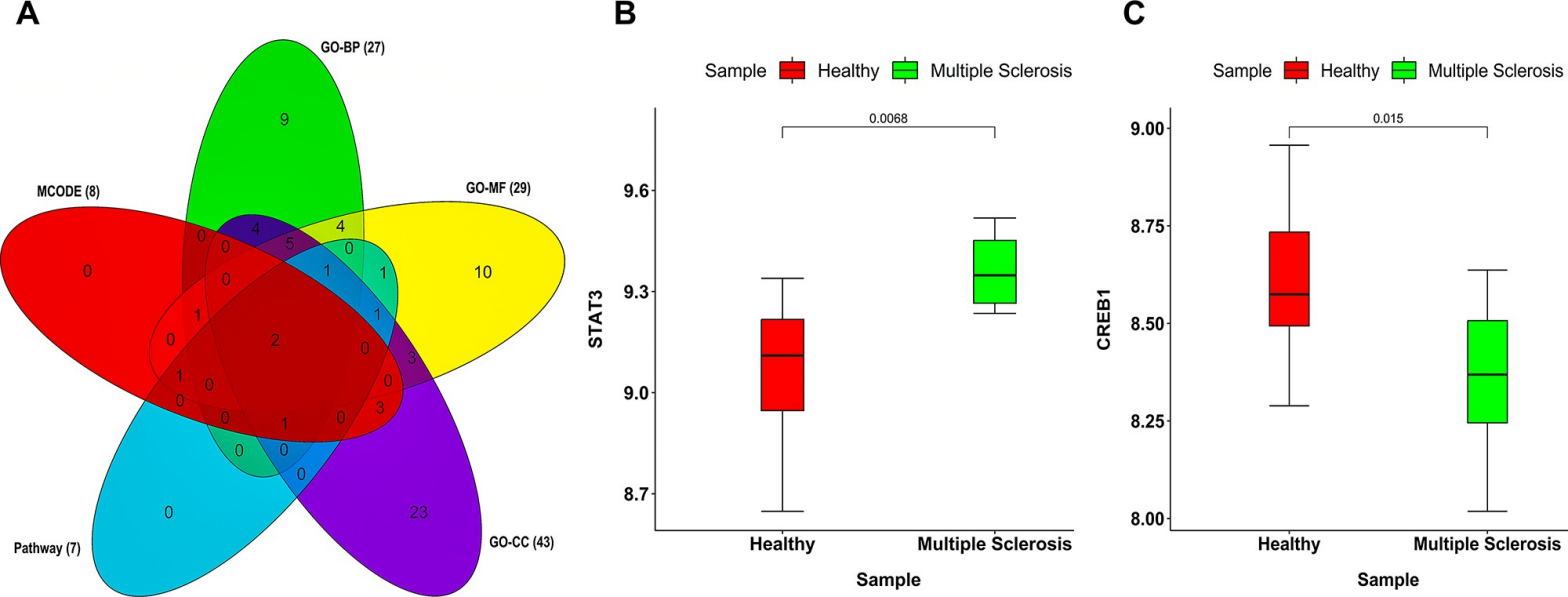
(A) Venn plot showing two overlapping hub DEG(s) between GO-BP, GO-MF, GO-CC, pathway, and MCODE top cluster genesets. Red, green, yellow, magenta, blue-colored areas signify MCODE, GO-BP, GO-MF, GO-CC, pathway genesets. Box-and-whisker plots showing the expression intensity distribution of (B) STAT3 and (C) CREB1 across MS and healthy patient samples. Red-and green-colored areas signify healthy normal and MS patient samples. The top and bottom of the boxes signify 75th and 25th percentile of distribution. Horizontal lines within the boxes represent the median values while the axes endpoints are labeled by minimum and maximum values.

Chord plots as shown in **Fig 3A–3D** shows the association of MS-associated hub DEGs with top 10 significant GO-BP, GO-MF, GO-CC, and pathway terms. Most significant pathway, GO-BP, GO-MF and GO-CC terms were assembly and cell surface presentation of N-methyl-D-aspartate (NMDA) receptors (p−value = 4.6×10^−4^), regulation of catabolic process (p−value = 3.1×10^−4^), NAD(P)H oxidase H_2_O_2_-forming activity (p−value = 5.4×10^−4^), postsynaptic recycling endosome (p−value = 3.0×10^−2^). Unweighted and undirected PPIN as shown in **Fig 4A** comprised 71 nodes and 72 edges corresponding to STRING interaction score > 0.4. Among them, 4 were upregulated while 67 were downregulated. MCODE revealed a total of 2 modules out of which module-1 (MCODE score =4.5) was considered as our PPIN hub module due to its top score. **Fig 4B** shows the top-scoring PPIN hub module comprising 5 nodes and 9 edges. Venn plot as shown in **Fig 5A** revealed signal transducer and activator of transcription 3 (*STAT3*) and *CREB1* as overlapping hub DEGs between GO-BP, GO-MF, GO-CC, pathway, and MCODE top cluster genesets. **Fig 5B and 5C** depicts box-and-whisker plots of the relative expression distributions of *STAT3* and *CREB1* across MS and healthy patient samples. As observed, *STAT3* is upregulated while *CREB1* is downregulated in case of MS samples compared to healthy normals. **Table 2** enlists the essential centrality measure values like ND, betweenness, closeness, and eigenvector for *STAT3* and *CREB1*. As observed from the table, *STAT3* had higher values of ND, betweenness, closeness, and eigenvector than CREB1; hence, we considered it our final biomarker.

**Table 2:** Degree, betweenness, closeness, and eigenvector values of hub DEGs within PPIN.

Gene	Degree	Betweenness	Closeness	Eigenvector
**STAT3**	8	0.55	0.40	0.43
**CREB1**	6	0.27	0.36	0.42

### 3.4. MS-associated 3-node miRNA FFL construction and topological analysis

**Fig 6:**
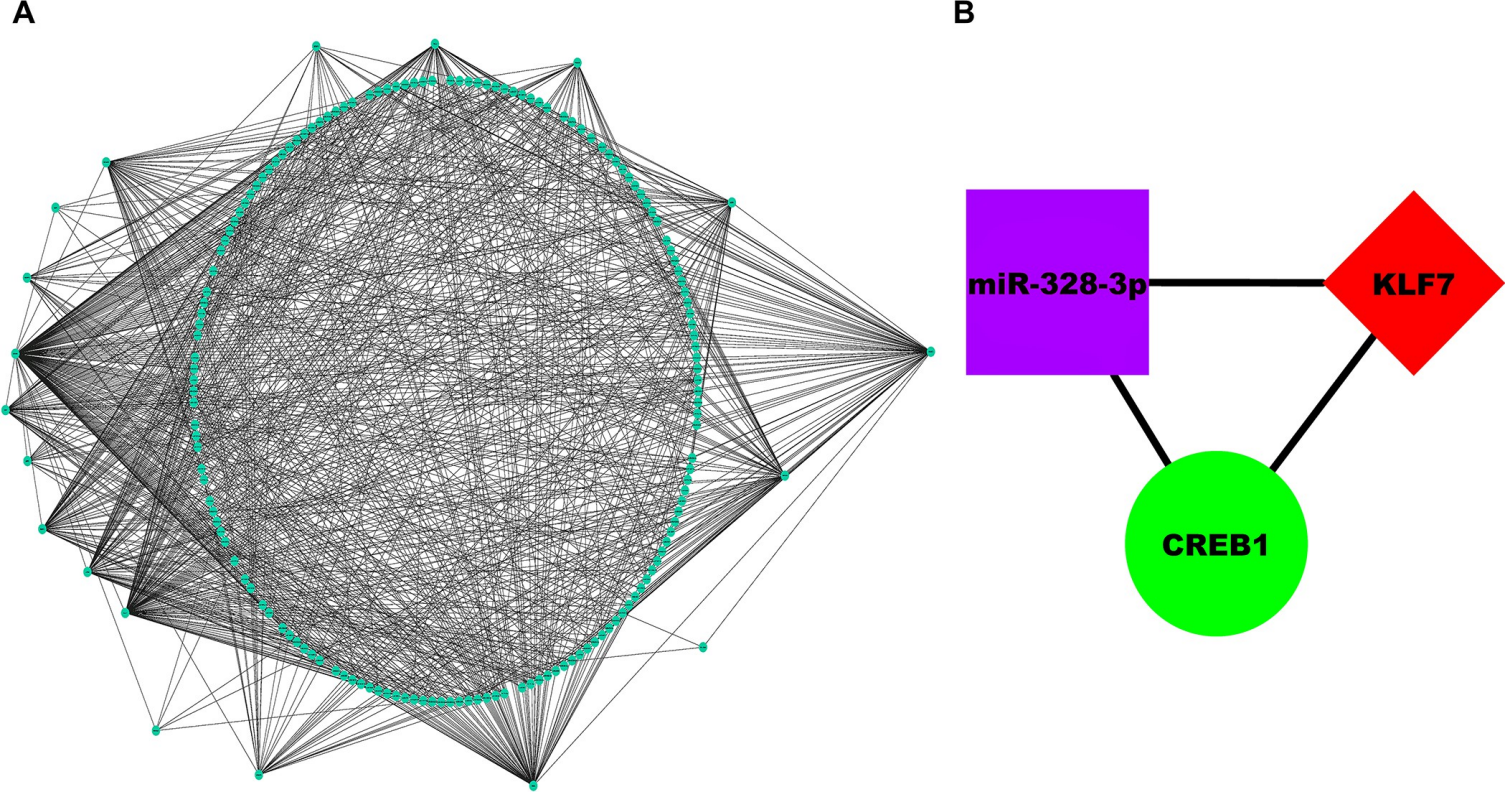
(A) Unweighted and undirected miRNA-mRNA-TF regulatory network comprising nodes and edges. (B) Highest-order subnetwork motif comprising one TF (KLF7), one miRNA (miR-328-3p), and one mRNA (CREB1). Magenta-colored rectangular nodes, green-colored circular nodes and red-colored diamond nodes signify miRNAs, mRNAs, and TFs, respectively.

**Fig 7:**
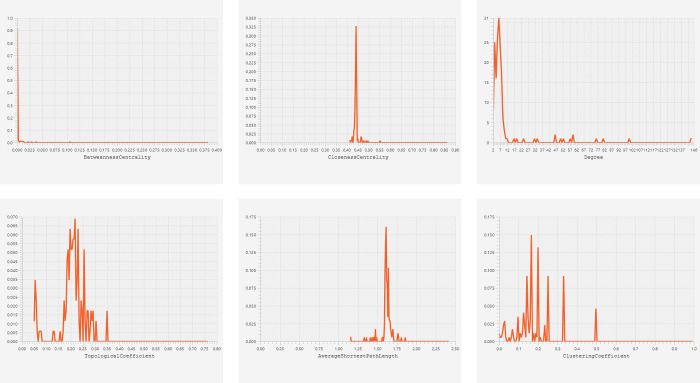
Topological/centrality distributions showing betweenness, closeness, node degree, topological coefficient, ASPL, and clustering coefficient of 3-node miRNA FFL.

MS-associated 3-node miRNA FFL as shown **Fig 6A** comprised 174 nodes and 895 edges. Among all edges, TF-mRNA pair constituted 28 edges, while miRNA-TF and miRNA-mRNA pairs constituted 672 and 195 edges, respectively. Among all nodes, 155,17,2 belonged to miRNAs, TFs, and mRNAs, respectively. Within this FFL, degree of TFs, miRNAs and mRNAs ranged from 3 to 101,2 to 12, and 77 to 146,respectively. The average degrees of TFs, miRNAs, and mRNAs were 41.17,5.59,111.5, respectively. **S2 Table** shows 3-node miRNA FFL nodes ranked based on betweenness, degree, and closeness. The table shows that miR-328-3p, *KLF7*, *CREB1* were the highest-ranked miRNA, TF, and mRNA based on these centralities and thus constitute the highest-order subnetwork motif within our FFL as shown in **Fig 6B**. **Fig 7** shows topological/centrality distributions like betweenness, closeness, ND, topological coefficient, average shortest path length (ASPL), and clustering coefficient of 3-node miRNA FFL.

## 4. Discussion

Assessment of gene expression data (GED) offers a valuable approach to pinpoint genes showing differential expression in disease conditions, providing potential insights into drug targets. This analysis allows researchers to comprehend the implicated BPs in disease and identify targets for intervention. In MS, abnormal gene expression, either overexpression or underexpression, significantly contributes to the disease’s onset and progression. For instance, the overexpression of proinflammatory genes intensifies IRs, causing myelin damage [44] and triggering autoimmune reactions [45–47]. Conversely, underexpression of anti-inflammatory genes impedes inflammation resolution, exacerbating tissue damage. Dysregulated gene expression also impacts immune cell activation, oligodendrocyte function, and neuroinflammation in MS. The expression heatmap illustrating the top 20 DEGs associated with MS reveals that the most upregulated gene is *CXCL10*, a chemokine, functioning as chemoattractants and proinflammatory modulators, plays a role in promoting the demyelination process [48]. Conversely, the most downregulated gene is *SLC38A11*, a member of the SLC38 family of transmembrane sodium-coupled amino acid transporters (AATs). These transporters are notably expressed in cells involved in significant amino acid metabolism (AAM) [49, 50]. This finding was also observed by *Hecker et al*. [51]. However, the specific role of *SLC38A11* in B cells and its relevance to MS remain unclear. Moreover, as observed from the box-and-whisker plots, there is an upregulation of *STAT3* and a downregulation of *CREB1* in MS samples compared to those from healthy individuals. Research findings indicate that there is abnormal STAT3 signalling in myeloid and lymphoid cells, such as neutrophils and CD4^+^ T cells in MS individuals [52]. Additionally, altered expression of genes related to axon guidance (AG) and synaptic plasticity (SP), particularly involving CREB signalling, has been highlighted via meta-analysis [53].

The transporter protein, SLC38A, involved in AAT, may modulate IRs by altering amino acid (AA) availability, which in turn influences immune cell function [54]. *CXCL10*, a chemokine, plays a critical role in MS by recruiting T cells to the CNS, contributing to the breakdown of the blood-brain barrier (BBB) and amplifying the inflammatory response [55]. *STAT3* is a TF activated by inflammatory signals such as C-X-C motif chemokine ligand 9 (*CXCL9*) and *CXCL10* [56–58], promotes the expression of genes involved in immune cell survival and inflammation, thus driving neuroinflammation in MS. *CREB1*, another TF, co-regulates inflammatory gene expression with *STAT3*, contributing to the IR and pathogenesis of MS [59]. Together, these molecules orchestrate a complex inflammatory network in MS. By visualizing the computational results, we hypothesize that *CXCL10* can activate *STAT3*, which in turn modulates *CREB1* activity, creating a feedback loop that amplifies inflammatory gene expression. *SLC38A11* may influence the overall energy and metabolic environment of immune cells, indirectly impacting the activation and regulation of *STAT3* and *CREB1*, thereby contributing to the pathogenic IR in MS. This intricate interplay suggests a coordinated regulatory network where metabolic and signalling pathways converge to drive neuroinflammation, highlighting potential therapeutic targets for modulating immune activity and mitigating disease progression in MS.

Furthermore, microarray technology is a widely utilized and powerful method for high throughput (HT) gene expression analysis and the study of DEGs in individuals with various disorders. It facilitates comprehensive exploration across pathway, GO-BP, GO-MF, and GO-CC terms, thus enhancing our understanding of complex biological networks. In the realm of MS research, the examination of significant pathways through these datasets has unveiled potential associations within PPIN or co-regulation networks. The GO-BP investigation yielded a list of potential PPINs or co-regulatory connections. Among these, the regulation of catabolic process emerged as the most statistically significant. Experimental research indicates that the catabolism of AAs is significantly reduced in individuals with MS due to elevation in proinflammatory cytokines and a decline in the number of Treg [60]. Particularly noteworthy are pathways involving the assembly and cell surface representation of NMDA receptors, signifying a focused exploration of the intricate processes governing the formation and localization of these essential receptors in the context of neural signalling. Besides, the GO-MF and GO-CC analysis highlights the NAD(P)H oxidase H_2_O_2_ -forming activity and postsynaptic recycling endosome (RE), providing valuable insights into oxidative stress (OS) pathways and SP. Studies indicate that the nuclei of dystrophic glia cells and neurons exhibit oxidized DNA, a condition where reactive oxygen species (ROS) cause damage to cellular components, including deoxyribonucleic acid (DNA) [61], linking oxidative damage to inflammation and an oxidative burst (OB). This process involves activated microglia and macrophages (MPs) expressing p22^phox^, a critical subunit of NADPH oxidases, further exacerbating the disease condition [62, 63]. Therapeutically, targeting NMDA receptors, modulating catabolic pathways for efficient debris clearance, and mitigating OS through interventions in NAD(P)H oxidase activity represent potential strategies for managing MS-related neurodegeneration and inflammation. Furthermore, the integration of MCODE cluster items further refines the identification and interpretation of functional modules within these intricate genetic landscapes (GLs).

The expression of mRNAs is regulated by miRNA and TFs, which tend to regulate each other as well. The interaction between these two factors creates two types of regulatory loops: FFLs and feedback loops (FBLs). FFLs involve a TF controlling the expression of a miRNA, while FBLs involve a miRNA suppressing a TF’s activity. These regulatory networks, particularly the FFL, play a crucial role in the pathological mechanisms of MS. FFLs consist of three interconnected nodes: a TF, its downstream miRNA, and a shared target mRNA. These FFLs are implicated in MS, where at least one of the three components can independently function as a proinflammatory or anti-inflammatory factor. In this context, we demonstrated that miR-328-3p, *KLF7*, and *CREB1* attained the highest rankings among miRNA, TF, and mRNA, respectively, based on their centrality measures. miR-328 has been identified as an inhibitor of neuron injury and inflammation [64]. Dysregulation of miR-328 was observed in both peripheral blood mononuclear cells (PBMCs) and brain white matter lesions (WMLs) of individuals with MS [23, 65, 66]. In the early stages of MS, active BLs are characterized by the presence of numerous inflammatory cells and MPs. In a study by *Yang et al*., the analysis of the miRNA-mRNA regulatory network revealed a significant upregulation of miR-328-3p in MS, consistent with findings from microarray analysis [67]. Consequently, our findings indicate that the foremost subnetwork motif involves a single TF (*KLF7*), a miRNA (miR-328-3p), and one mRNA (*CREB1*), as illustrated in **Fig 6B**. Meanwhile, the TF *KLF7* is known for its role in regulating axon outgrowth [68]. In instances of CNS and peripheral nerve injury (PNI), the upregulation of *KLF7* has been shown to promote growth, axonal regeneration, and sprouting in damaged nerves [25, 69, 70]. Additionally, TFs of the CREB family, crucial for axonal regeneration, plasticity, cell survival, and neuroprotection [71], have been reported to be dysregulated in lesions from mixed MS brain tissue [53, 72, 73]. Studies suggest that dysregulated CREB signalling, as indicated by the neuronal methylome, may be associated with neuro-axonal impairment in MS [53]. The interplay between miR-328, *KLF7*, and CREB signalling pathways underscores their potential roles in the complex mechanisms contributing to neuron injury, inflammation, and impaired neuro-axonal function observed in MS. This network of TF-miRNA-mRNA loops may contribute to a deeper understanding of the mechanisms behind MS as well as the identification and screening of therapeutic targets for potential pharmaceutical applications. Further research in this area may provide insights into potential therapeutic targets for managing MS-related neurodegenerative processes.

## 5. Conclusion

MS is a multifaceted neurological disorder witnessing a significant surge in prevalence. Our research reveals *STAT3* and *CREB1* as biomarkers playing an essential role in MS progression dynamics via a combined multiomics and network-based approach. *STAT3* and *CREB1* expression levels were significantly up and downregulated, respectively, in MS patients as compared to healthy normals. The sequential flow of transcriptomics analysis, WGCN hub DEGs selection, functional enrichment, PPIN modular analysis, and FFL analysis gave final robust key elements as *KLF7*, miR-328-3p, and *CREB1* jointly working in a coordinated fashion to manage MS complex molecular mechanism. Further experimental studies are needed to validate their importance in MS progression and thereby may prove to be diagnostic biomarkers for early detection and therapeutics.

## Supporting information

S1 File (XLSX)

S1 Table List of MS-associated DEGs. (XLSX)

S2 Table Essential centrality/topological properties of top-ranked miRNAs/mRNAs/TFs within 3-node miRNA FFL. (XLSX)
